# Immune modulation by molecularly targeted photothermal ablation in a mouse model of advanced hepatocellular carcinoma and cirrhosis

**DOI:** 10.1038/s41598-022-15948-3

**Published:** 2022-08-24

**Authors:** Nina M. Muñoz, Crystal Dupuis, Malea Williams, Katherine Dixon, Amanda McWatters, Jie Zhang, Swathi Pavuluri, Arvind Rao, Dan G. Duda, Ahmed Kaseb, Rahul A. Sheth

**Affiliations:** 1 Department of Interventional Radiology, The University of Texas MD Anderson Cancer Center, T. Boone Pickens Academic Tower (FCT14.5092), 1515 Holcombe Blvd., Unit 1471, Houston, TX 77030 USA; 2 Department of Experimental Radiation Oncology, The University of Texas MD Anderson Cancer Center, Houston, TX USA; 3 Department of Computational Medicine and Bioinformatics; Department of Radiation Oncology, University of Michigan, Ann Arbor, MI USA; 4 Edwin L. Steele Laboratories for Tumor Biology, Department of Radiation Oncology, Massachusetts General Hospital, Boston, MA USA; 5 Department of Gastrointestinal Medical Oncology, The University of Texas MD Anderson Cancer Center, Houston, TX USA

**Keywords:** Cancer, Liver cancer

## Abstract

Immunotherapy is a promising new treatment approach for hepatocellular carcinoma (HCC), but there are numerous barriers to immunotherapy in HCC, including an immunosuppressive microenvironment and the “immunotolerance” of the liver. Hyperthermia treatment modalities are standard of care for early stage HCC, and hyperthermia is known to have immunomodulatory effects. We have developed a molecularly targeted photothermal ablation (MTPA) technology that provides thermally tunable, tumor-specific heat generation. The purpose of this study was to evaluate the morphologic and immunologic effects of MTPA in an immunotherapy-resistant syngeneic mouse model of HCC in a background of toxin-induced cirrhosis. We found that the anatomic, cellular, and molecular features of this model recapitulate the characteristics of advanced human HCC. MTPA as a monotherapy and in combination with immune checkpoint therapy significantly increased intratumoral CD3+ and activated CD8+ T cells while decreasing regulatory T cells relative to control or immune checkpoint therapy alone based on immunohistochemistry, flow cytometry, and single cell RNA sequencing data. Furthermore, we identified evidence of MTPA’s influence on systemic tumor immunity, with suppression of remote tumor growth following treatment of orthotopic tumors. The results of this study suggest that tumor-specific hyperthermia may help overcome resistance mechanisms to immunotherapy in advanced HCC.

## Introduction

Hepatocellular carcinoma (HCC) is the most common type of primary liver cancer and the third most common cause of cancer-related death worldwide^1^. For patients with small, solitary HCC lesions, local tumor control with hyperthermia modalities such as radiofrequency ablation (RFA) is highly effective. However, the vast majority of patients with HCC present with advanced stage disease, a diagnosis with few treatment options. This limited toolkit has broadened substantially in the past half-decade with the demonstrated clinical benefit of immune checkpoint inhibitor-based immunotherapies. Immunotherapy has revolutionized cancer care, and recently it has been shown to be effective in HCC^2^. Phase I/II trials of anti-PD-1 and anti-CTLA-4 checkpoint inhibitors for HCC have demonstrated treatment responses; unfortunately, though, the response rates remain low^3,4,5,6,8^. For example, the conducted CheckMate 040 trial, a phase I/II study of an anti-PD-1 checkpoint inhibitor for patients with advanced HCC, demonstrated an objective response rate of only 20% in the dose-expansion cohort^9^. Likewise, a phase III trial comparing the PD-1 inhibitor pembrolizumab to placebo failed to meet its prespecified statistical significance^10^. A major question in HCC immunotherapy, therefore, is how to address treatment resistance and broaden its benefits to a greater proportion of HCC patients.

One promising treatment approach is to combine locoregional therapies such as hyperthermia with systemic immunotherapies. As hyperthermia is known to have immunomodulatory effects^11,12^, a combination-based strategy has the potential to overcome barriers to systemic immunotherapies. Clinical implementation of such treatment paradigms requires pre-clinical validation, though, for which animal models play a key role. For an animal model to faithfully recapitulate the human disease, it must accurately replicate the key features of HCC’s immune microenvironment, namely, a baseline resistance to monotherapeutic checkpoint inhibition in a background of chronically inflamed cirrhotic liver parenchyma. Moreover, a model’s similarity with the molecular underpinnings that drive HCC’s immune evasion^13^ is also critical to evaluate methods to overcome these barriers.

The purpose of this study was to investigate the anatomic, cellular, and molecular features of an immunotherapy-resistant syngeneic mouse model of HCC, with both orthotopic and subcutaneous tumors, in a background of toxin-induced cirrhosis. We evaluated the morphologic and immunologic characteristics of this model at baseline as well as following immune checkpoint inhibitor therapy. We also assessed the immunologic response in this model to a tumor-specific hyperthermia modality that we have previously developed termed molecularly targeted photothermal ablation (MTPA)^14^ and its ability to overcome resistance to immune checkpoint inhibitor therapy.

## Results

### Characterization of a syngeneic, orthotopic, immunocompetent mouse model of HCC in a background of cirrhosis

**Figure 1:**
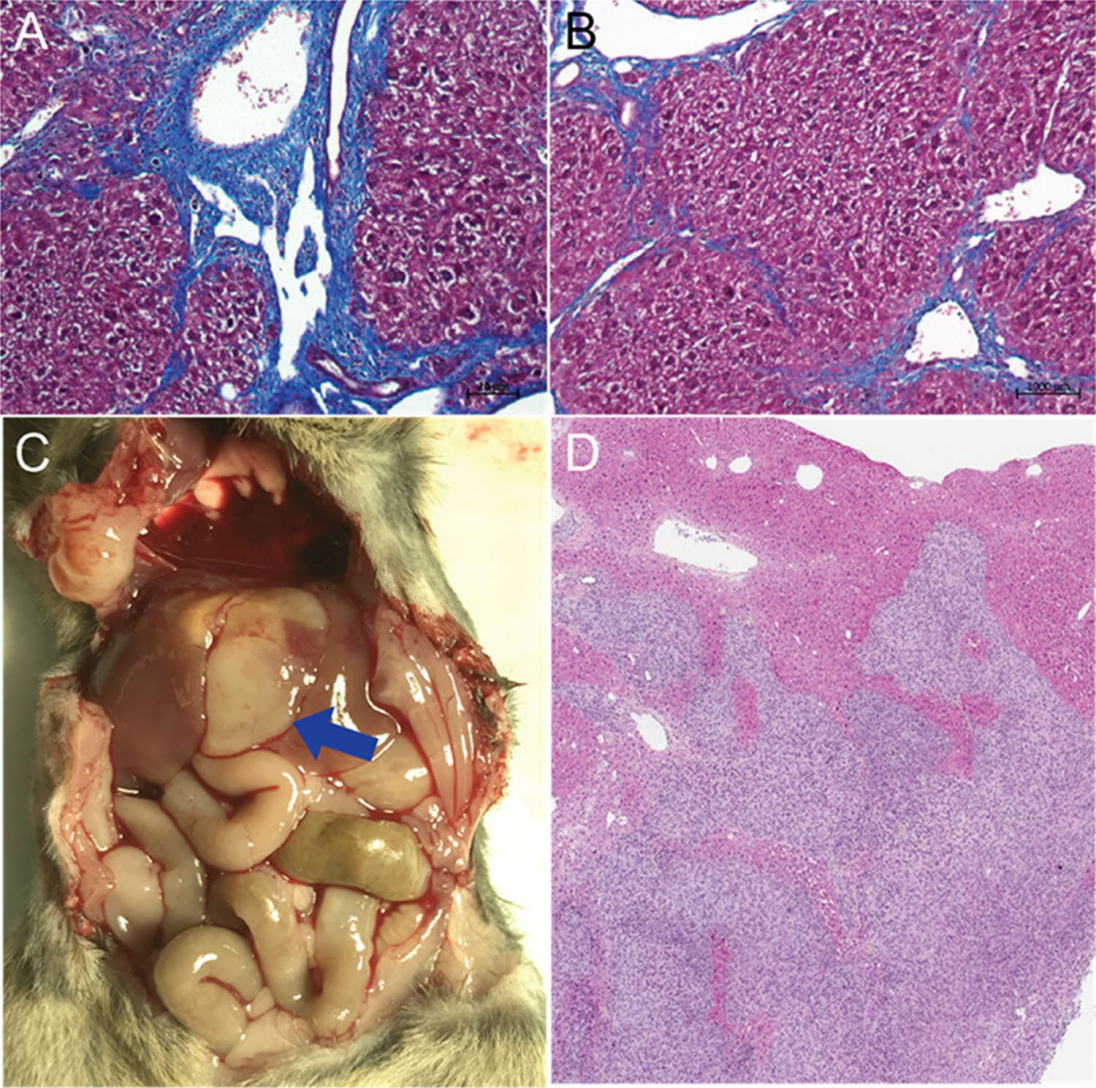
Characterization of syngeneic murine HCC tumor model with cirrhosis. (**A**, **B**) Trichrome staining of liver tissue following 12 weeks of oral gavage of carbon tetrachloride demonstrates periportal and bridging fibrosis, recapitulating the histologic findings in human cirrhosis. (**C**, **D**) Orthotopic tumor implantation (blue arrow) exhibits a high rate of engraftment as well as a diffuse, infiltrative phenotype, a pattern commonly seen in aggressive hepatocellular carcinoma in patients.

**Figure 2:**
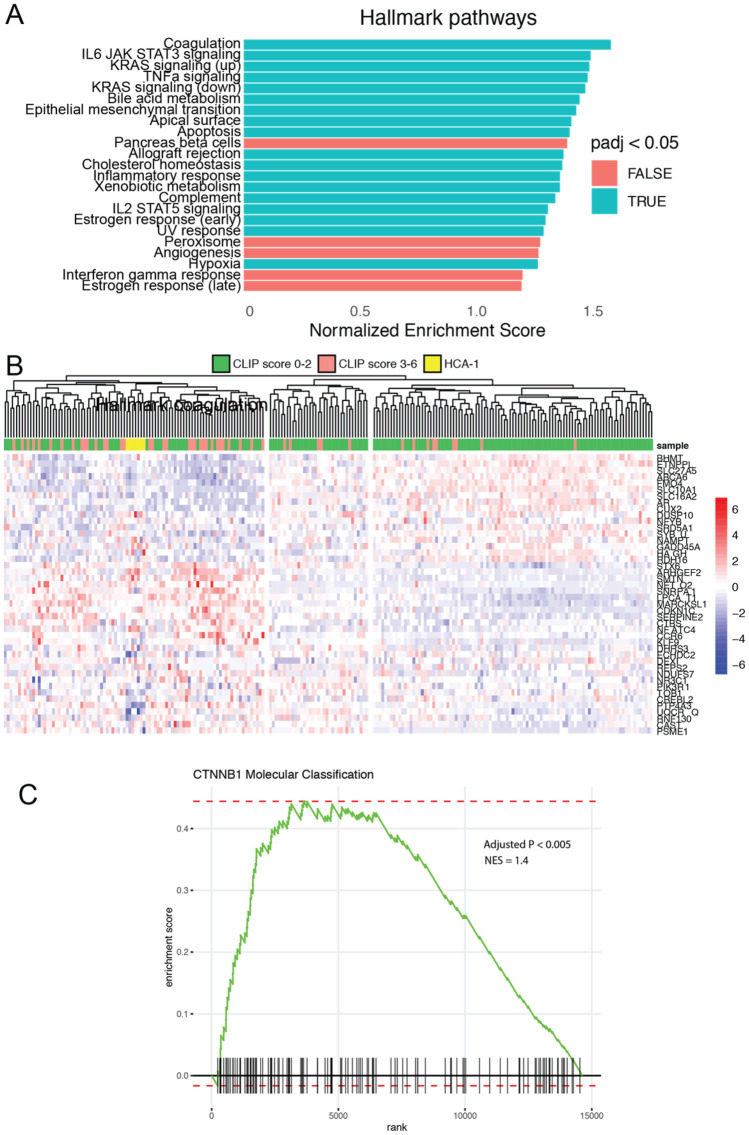
Genomic analysis of cirrhosis and orthotopic HCC tumors. (**A**) Transcriptome data of cirrhotic mouse tissue acquired by RNAseq were compared against the Hallmark gene sets (MSigDB), highlighting upregulation of numerous growth factor and inflammatory pathways (e.g., IL6, KRAS, TNFα) commonly seen in human cirrhosis. (**B**) Transcriptome data of orthotopic HCA-1 mouse tumor tissue acquired by RNAseq were compared to previously published human HCC tumors (Gene Expression Omnibus accession GSE14520); mouse tumors were found to cluster with advanced human HCC tumors (CLIP score > 2). (**C**) Gene set enrichment analysis of the HCA-1 cell line transcriptome revealed a strong similarity with the previously published gene expression profile for HCC tumors classified by gain-of-function mutations of *CTNNB1*, a molecular subclassification characterized by an “immune desert” phenotype.

We first evaluated the morphologic, histologic, and molecular features of a murine model of HCC generated by the orthotopic implantation of a syngeneic hepatoma cell line following the induction of cirrhosis (Figs. 1 and 2). Trichrome staining of the liver parenchyma confirmed the development of bridging fibrosis, a key feature of human cirrhosis. Transcriptomic evaluation of the cirrhotic tissue using bulk RNAseq revealed upregulation of numerous hallmark gene pathways that are commonly associated with human cirrhosis. These included the interleukin-6 (IL-6) pathway, the KRAS pathway, and the TNFα pathway. Histologic evaluation of orthotopically implanted hepatoma cells illustrated an infiltrative phenotype commonly seen in high-grade human HCC.

We then performed transcriptome analysis of orthotopic HCC tumors. First, we performed gene set enrichment analysis and found a strong correlation between the HCC model’s gene expression profile with that of a previously published gene set defining the clinical molecular subtype of HCC characterized by gain-of-function mutations in the *CTNNB1* (β-catenin) gene (NES = 1.4, *P* < 0.005). We also compared the mouse model’s transcriptional profile against gene expression data from a large human data set to determine how the mouse model compared across a range of HCC tumors with known clinical staging. Unsupervised clustering was performed using the top 50 differentially expressed genes from the human HCC data, and a heatmap was generated for visualization. Clustering revealed three principal clusters within the human dataset; the greatest proportion of high-grade HCC tumors (CLIP score > 2) localized to the first cluster, while the greatest proportion of low-grade HCC tumors (CLIP score < 2) localized to the third, corresponding to a transcriptomic pattern of high (cluster 1), intermediate (cluster 2), and low (cluster 3) grade tumors. The mouse HCA-1 tumors localized to the high-grade cluster (cluster 1), indicating the model’s similarity to aggressive human HCC tumors.

### MTPA is a clinically translatable, thermally tunable ablation modality

**Figure 3:**
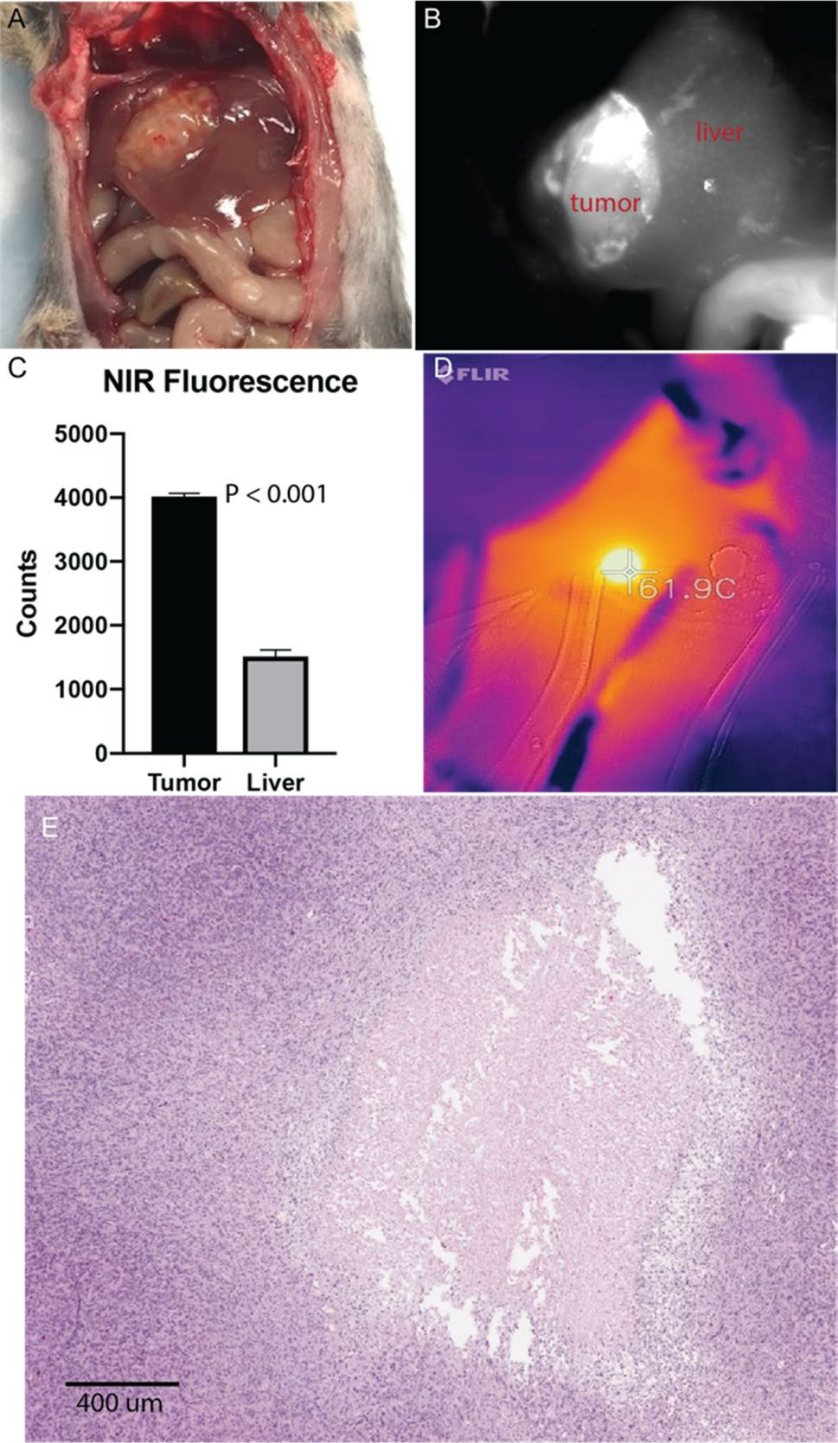
Molecularly targeted photothermal ablation (MTPA) of orthotopic HCC tumors. Mice with orthotopic tumors (**A**) were administered 0.5 mg/kg ICG via tail vein. MTPA was performed 6 h following injection. Near infrared imaging during the intervention demonstrated excellent target-to-background ratios (**B**, **C**). Temperature monitoring was performed with real-time thermal imaging. Hyperthermia up to ablative temperatures were readily achievable, and thermal doses could be tuned by adjusting the illuminating laser power (**D**). MTPA resulted in targeted necrosis within the orthotopic tumor (**E**).

We confirmed the ability of MTPA to generate tumor-specific heat based upon the localization of ICG to HCC tumors in this mouse model (Fig. 3). Following the intravenous administration of 0.5 mg/kg ICG, we performed MTPA by illuminating liver tumors 785 nm, 400mW NIR light at 6 h post ICG injection (n = 10). Hyperthermia developed within the tumors, and the hyperthermia was found to be tunable by adjusting the laser power (0–400 mW), with temperatures ranging from 37 °C to > 100 °C.

### MTPA modifies the tumor immune microenvironment towards an immunologically “hot” state

**Figure 4:**
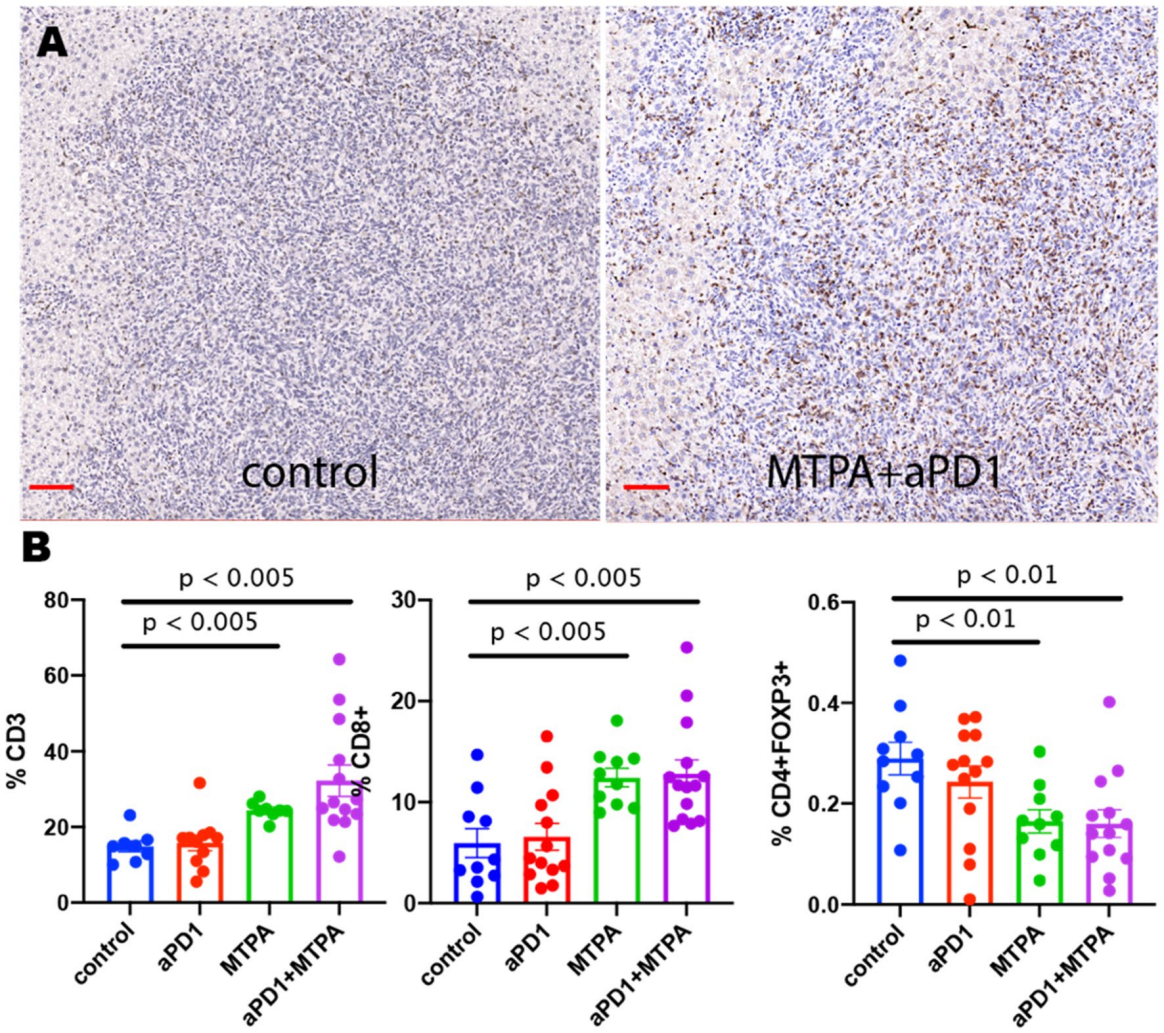
Immunohistochemical analysis of orthotopic HCC tumors treated with MTPA. MTPA was performed in an orthotopic mouse model of HCC with underlying cirrhosis. (**A**) IHC of CD8+ cells demonstrates minimal infiltrating T cells in control tumors but a substantial increase following anti-PD1 therapy combined with MTPA (red bar = 100 mm). (**B**) Quantitative analysis of IHC data (error bars reflect standard error) reveals a significant increase in intratumoral CD3+ and CD8+ T cells following MTPA alone and MTPA+anti-PD1 therapy, along with a decrease in CD4+FOXP3+ T_regs_. Percentages in panel B reflect the proportion of positively stained cells within a region of interest circumscribing the tumor. Box plots reflect mean +/−  standard error. The non-parametric Mann–Whitney U-test was used to evaluate differences in the proportions across treatment groups.

**Figure 5:**
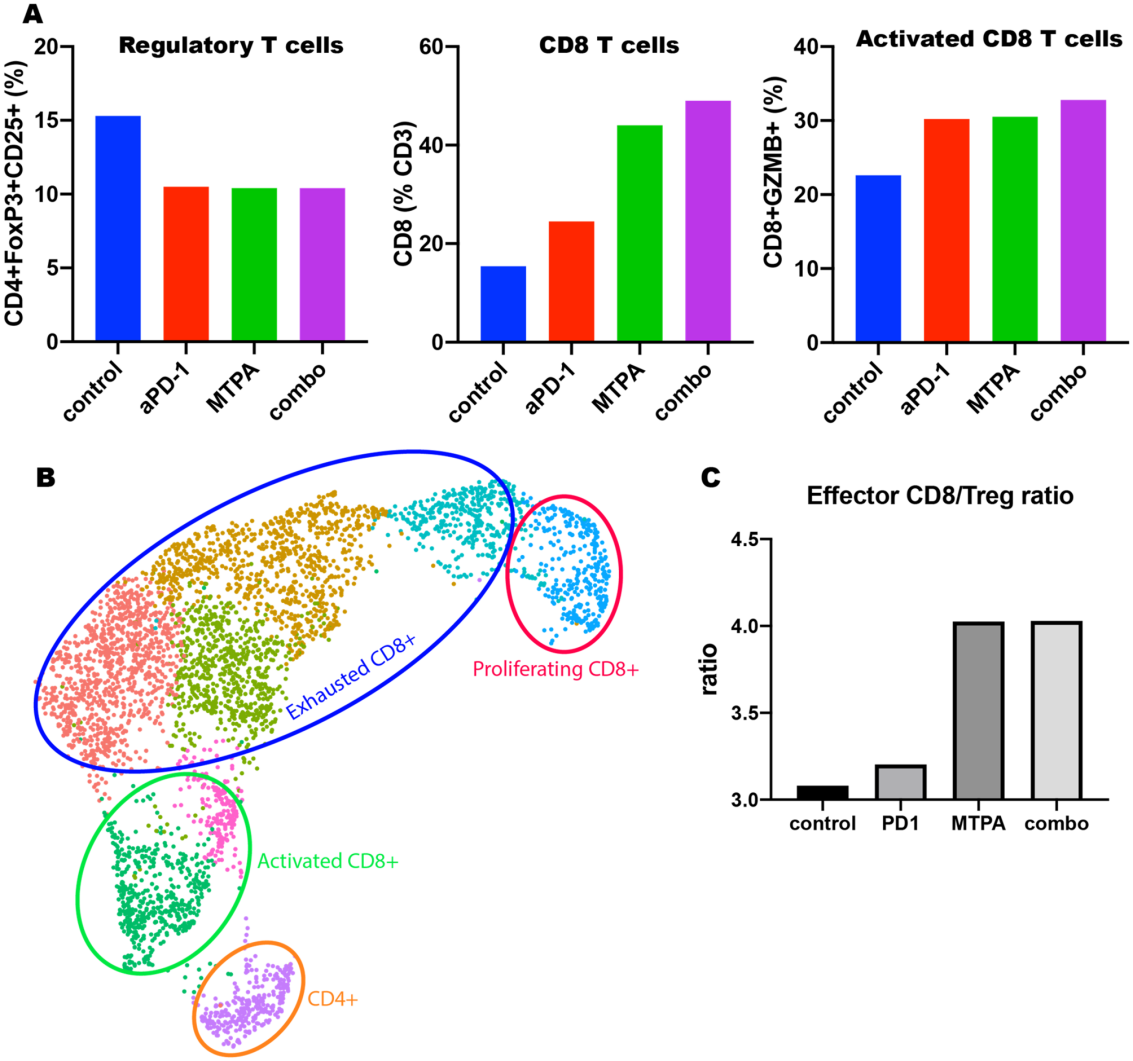
MTPA augments tumor immunity. MTPA +/− anti-PD-1 therapy was performed in an orthotopic mouse model with underlying cirrhosis. A, flow cytometry demonstrated an increase in the overall CD8 T cell population as well as activated effector CD8 T cells within the tumors treated with MTPA and combination therapy; there was also a concomitant decrease in the T_reg_ population in these tumors. B, C, These findings were confirmed on scRNAseq of intratumoral T cells highlighted a significant increase in the ratio of activated effector CD8+ T cells to immunosuppressive T_regs_ with MTPA but not with anti-PD-1 monotherapy.

We next found that MTPA has potent immunostimulatory effects on the tumor immune microenvironment in this treatment resistant mouse model of HCC (Figs. 4 and 5). Cirrhotic mice with orthotopic tumors (n = 12 per arm) were randomized to receive systemic anti-PD-1 therapy + sham surgery, anti-PD-1 therapy + MTPA, MTPA alone, or sham surgery. On POD 7, orthotopic tumors were harvested, and immunohistochemical staining for immune cell populations was performed. This analysis revealed a significant increase in intratumoral CD3+ and CD8+ T cells following MTPA alone and MTPA+anti-PD1 therapy, along with a decrease in CD4+FOXP3+regulatory T cells (T_regs_). These findings were corroborated by flow cytometry data that revealed an increase in overall CD8+ T cells (control 15.4%, anti-PD1 therapy 24.5%, MTPA 44%, MTPA+anti-PD1 therapy 49%) as well as the subpopulation of activated CD8+ T (control 22.6%, anti-PD1 therapy 30.2%, MTPA 30.5%, MTPA+anti-PD1 therapy 32.8%) cells following MTPA and MTPA+anti-PD1 therapy compared to control or anti-PD1 therapy alone. Likewise, there was a decrease in T_reg_ cells (control 15.3%, anti-PD1 therapy 10.5%, MTPA 10.4%, MTPA+anti-PD1 therapy 10.4%) in MTPA and MTPA+anti-PD1 therapy arms relative to control or anti-PD1 therapy alone.

Furthermore, single cell RNA sequencing (scRNAseq) was performed on the intratumoral CD3+ T cell population. The ratio of activated effector T cells to immunosuppressive regulatory T cells (Tregs) was minimally different with anti-PD-1 therapy (3.2 vs 3.1, 3% change from sham) alone compared to sham, highlighting the challenges of immunotherapy as a monotherapy in this mouse model. The addition of MTPA significantly increased this ratio (4.0 vs 3.1, 29%), emphasizing the potential role of MTPA as an adjuvant intervention to modify the tumor microenvironment. Interestingly, however, there was no further improvement in the ratio when anti-PD-1 therapy was combined with MTPA (4.0 vs 3.1, 29%).

**Figure 6:**
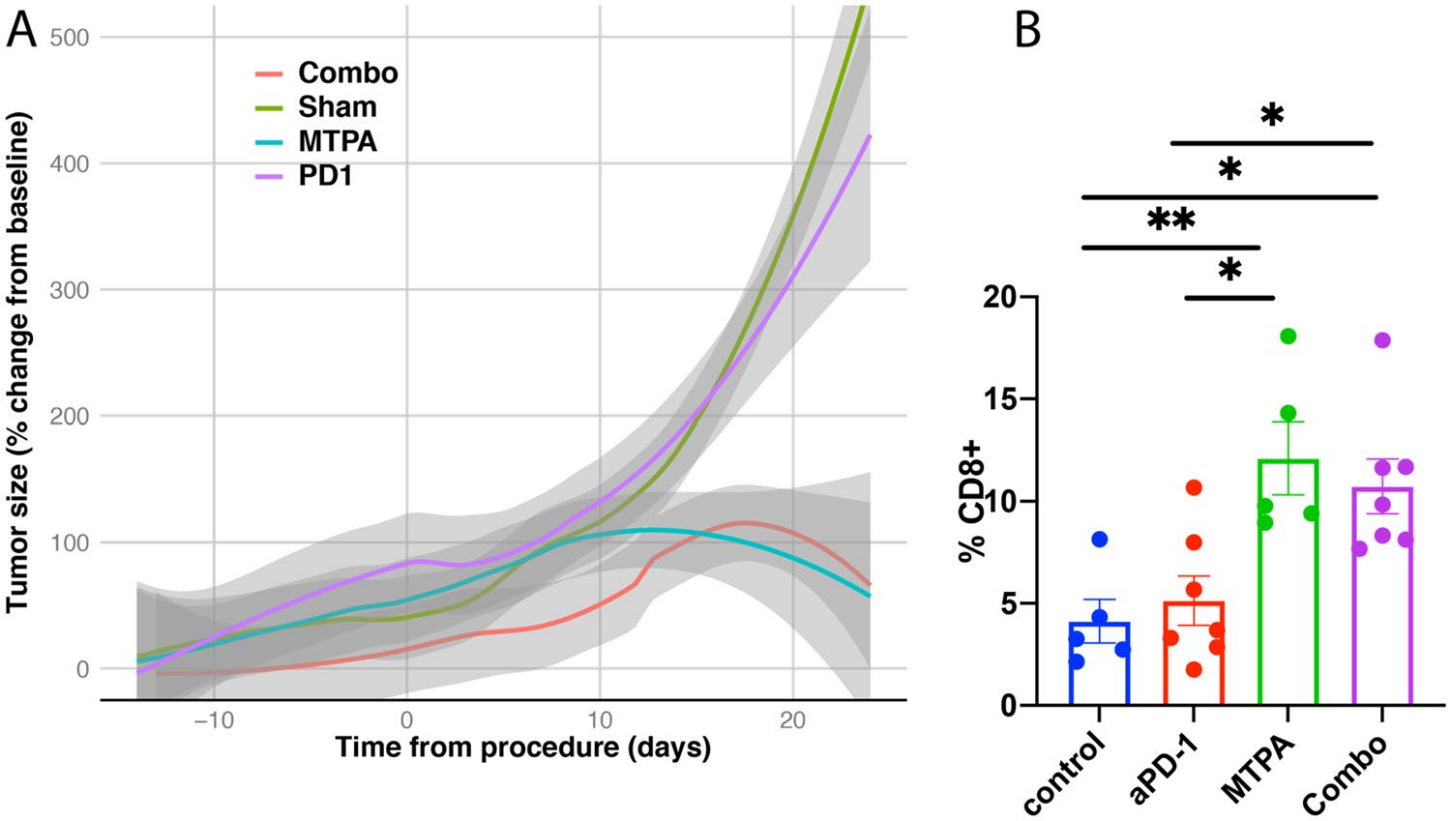
Growth response of remote tumors following MTPA to orthotopic HCC tumors. (**A**) Remote tumors demonstrated continued growth following sham surgery (control arm, n = 15) or anti-PD-1 therapy (n = 12). However, there was inhibition of remote tumor growth following MTPA treatment to the orthotopic liver tumor (n = 12) and in the combination MTPA+anti-PD-1 therapy (n = 12), though there was no difference identified between the MTPA alone versus the combination arm. (**B**) Immunohistochemical analysis demonstrated a significant increase in infiltrating CD8+ T cells within the subcutaneous tumors when the orthotopic liver tumor was treated with MTPA and MTPA+anti-PD-1 therapy relative to sham surgery and anti-PD-1 therapy alone (*P < 0.05; **P < 0.01). Percentages in panel B reflect the proportion of positively stained cells within a region of interest circumscribing the tumor. Box plots reflect mean +/− standard error. The non-parametric Mann–Whitney U-test was used to evaluate differences in the proportions across treatment groups.

Likewise, we identified evidence of MTPA’s influence on systemic tumor immunity, also known as “abscopal” effect. To this end, both orthotopic and subcutaneous tumors were implanted in mice, and the animals were treated with either sham surgery (n = 15), systemic anti-PD-1 therapy (n = 12), local MTPA (n = 12), or MTPA+anti-PD-1 therapy combination (n = 12) to the orthotopic tumor. Growth curves from the subcutaneous tumor (Fig. 6) reveal unabated growth in the sham surgery and anti-PD-1 therapy arms. However, there was inhibition of tumor growth and regression following MTPA and the combination arm, though the addition of anti-PD-1 therapy did not appear to add additional benefit to MTPA alone.

## Discussion

In this study, we present the immune profiling of an orthotopic, syngeneic mouse model of HCC with cirrhosis. The underlying cirrhosis displays numerous transcriptional features of human cirrhosis, and the orthotopic tumors exhibit molecular and morphologic features similar to that of aggressive HCC tumors in humans. As with the human disease, the model demonstrates intrinsic resistance to single agent immunotherapy, but immunomodulatory interventions such as MTPA can generate immunogenic changes to local and systemic tumor immunity.

Animal models have been central to the study of liver cancer and the basis for preclinical and translational research of HCC. Unfortunately, it has been difficult to generate a mouse model that can be used universally due to the genetic heterogeneity of liver cancer. Moreover, HCC is differentiated from most other cancers by the nearly ubiquitous comorbidity of cirrhosis. The presence of cirrhosis is critical for the validity of an animal model of HCC^15^. Likewise, orthotopic tumors more accurately replicate the tumor immune microenvironment and are necessary for immunologic studies^15,16^. Alternative animal models that feature cirrhosis and are commonly used, particularly for the study of locoregional therapies, are the diethylnitrosamine (DEN)-induced autochthonous rat HCC model^17^ and the woodchuck hepatitis virus-induced woodchuck HCC model^18^. While tumors develop in a background of chronic inflammation in these models, a substantial limitation is the lack of species-specific immunologic assays as well as the unavailability of species-specific immune checkpoint inhibitors. Given the preponderance of immunologic assays available for mice, as well as the availability of multiple checkpoint inhibitor therapies, there are numerous practical advantages to mouse models of HCC.

There is a robust scientific premise for the role of immunotherapy in HCC. Increased expression of PD-L1 in HCC tumors portends a poorer prognosis, and the level of PD-L1 expression is an independent predictor of HCC recurrence following liver resection surgery^19^. Clinical trials evaluating single agent checkpoint inhibition for HCC showed signs of efficacy, though only for a minority of patients^20^. Combination regimens, on the other hand, have shown improved outcomes. For example, ipilimumab (anti-CTLA4) in combination with nivolumab (anti-PD1) is an approved second-line therapy for HCC. Furthermore, combination strategies that pair immunotherapy with drugs that modulate the tumor microenvironment have recently substantially advanced the field of systemic therapies for advanced HCC. The most notable of these new regimens is the combination of an anti-PD-L1 antibody with an anti-angiogenesis drug^21^; this is now a standard of care first-line therapy.

Tumor genomics plays a key role in driving immune evasion. For HCC, as with other malignancies, gain-of-function mutations in the *CTNNB1* (β-catenin) gene have recently been shown to strongly impact the tumor immune microenvironment^19^. This molecular subclassification of HCC is characterized clinically by T cell exclusion and resistance to anti-PD-1 inhibition therapy^20,22^. We found that the animal model investigated in this study correlates well with subtype of HCC, not only in its gene expression profiling but also in its lack of infiltrating T cells and resistance to checkpoint inhibition. We also found, though, that hyperthermia by MTPA could overcome these barriers to tumor immunity.

Hyperthermia is well known to counteract an immunosuppressive microenvironment. The immune response to hyperthermia is dependent on the thermal dose and thus impacted by both temperature and duration. “Fever” range heating (37–41** °**C) does not result in direct cell death, but it can increase APC activity and improve co-stimulatory molecule presentation to T cells^23,24^. It can also increase expression of MICA, an NKG2D ligand, thus increasing the efficacy of natural killer cells^25^. Fever range heating also improves immune cell trafficking by increasing ICAM-1 expression on tumor vasculature^26^. In sublethal hyperthermia (42–47 °C), HSPs are unable to reverse thermally induced protein denaturation but are effective chaperones of tumor neoantigens to APCs, promoting anti-tumor adaptive immunity. Heating in this temperature range also increases MHC class I expression levels, allowing improved recognition of tumor cells by CD8+ T cells^27^. Ablative hyperthermia (> 47** °**C) results in direct coagulative necrosis and through the release of HSPs can activate an adaptive immune response. However, the obliteration of vasculature, the loss of tumor neoantigens through protein denaturation, and the release of oncogenic inflammatory cytokines may explain why a sustained anti-tumor response is rarely seen after conventional ablation procedures. Indeed, conventional thermal ablation modalities such as radiofrequency ablation have been shown to cause distant tumor growth preclinical liver tumor models^28–30^. The driving force behind this ablation-mediated oncogenesis is the appropriation by tumor cells of the regenerative wound healing response triggered by the thermally injured liver parenchyma. Upon exposure to hyperthermia, hepatocytes upregulate a host of inflammatory cytokines including TNFα and IL-6^31,32^ that can drive inflammation-mediated carcinogenesis and result in local and remote tumor growth^28,33^.

Thus, optimizing hyperthermia for anti-tumor immunity requires not only identification of an appropriate thermal dose but also ensuring that the hyperthermia is confined to the tumor itself, with minimal extension into the adjacent hepatic parenchyma. We have previously shown that MTPA generates tumor-specific hyperthermia and results in diminished systemic release of oncogenic cytokines relative to conventional ablation modalities in a rat model of HCC^14^. We have also demonstrated previously that MTPA decreases β-catenin expression levels, potentially providing a mechanism for the subsequent microenvironmental changes. In this study, we likewise identified immunogenic modulations to the tumor immune microenvironment and systemic tumor immunity in a murine model that more faithfully recapitulates the human disease given the background of cirrhosis. An important limitation of this study, however, is the fact that we did not evaluate for immunologic “memory” by implanting tumors following treatment of the orthotopic tumor. It is also important to note that in addition to hyperthermia, transarterial embolization is a cornerstone in the management of HCC. Recent studies, such as those by Tischfield et al.^34^, have investigated the immunologic ramifications of this locoregional therapy. In this study, the authors found that embolization performed on autochthonous tumors in a rat HCC model resulted in increased infiltration of lymphocytes as well as PD-L1 expression, a finding consonant with the results of this study.

This study also evaluated the influence of MTPA on immune checkpoint blockade therapy for HCC. This approach has previously been evaluated in early-stage clinical trials. For example, in a trial of 39 patients with intermediate and advanced stage HCC, patients underwent ablation or trans-arterial therapies in combination with tremelimumab (anti-CTLA)^35^. The combination was safe, with immune profiling demonstrating a significant increase in infiltrating T cells for patients who exhibited clinical response. Given the single arm nature of the study, though, it is unknown whether the T cell infiltration was principally driven by the locoregional therapy versus the checkpoint inhibitor. Nevertheless, given the potential for local therapies to overcome barriers to immunotherapy, there are numerous similar combination studies currently underway for HCC^18^. Interestingly, though, we did not find evidence for combinatorial gains when anti-PD-1 therapy was added to MTPA. One possible explanation is that the PD-1 pathway is not the most appropriate target for this combinatorial approach. That is, while the data on hyperthermia + PD-1 inhibition is still nascent, there have been numerous studies combining other local therapies such as radiation with PD-1 inhibition, many of which have failed^36^. Furthermore, we have previously shown that hyperthermia activates multiple immune checkpoint pathways in tumors, including PD-1 as well as CTLA-4 and VISTA^14^. Thus, hyperthermia itself may raise immunosuppressive barriers that require an immune checkpoint inhibitor “cocktail” to overcome. Identifying the optimal combination regimens for locoregional therapies and immune checkpoint inhibitors will be essential to improve the efficacy of immune-mediated therapies for HCC.

## Materials and methods

### Animal model of HCC in background of cirrhosis

All animal experiments were approved by The University of Texas, MD Anderson Cancer Center’s Institution Animal Care and Use Committee. All methods were performed in accordance with the ARRIVE guidelines and in accordance with the relevant guidelines and regulations. Unless otherwise indicated as a replicate measurement, data were taken from distinct samples. The generation of a syngeneic mouse model of HCC in a background of cirrhosis was performed according to previously published techniques^37^. In brief, C3H mice (Charles River) were administered 150 µL of CCL_4_ by oral gavage three times weekly for 12 weeks to generate liver fibrosis. Following this, orthotopic and/or subcutaneous tumor implantation was performed in the following manner. HCA-1 murine hepatoma cell line, the orthotopic HCC model, and the response to immunotherapy were applied and evaluated as previously described^38^. The cell line was shown to be negative for mycoplasma or other contaminants by MAP testing (PCR method). Cells were cultured in antibiotic-free, sterile, high-glucose DMEM medium (Gibco, cat. 11965-092), supplemented with 10% (vol/vol) FBS for two weeks prior to implantation. The HCA-1 cells were seeded at a density of 2 × 10^6^ on a T-175 cm^2^ flask 2 days prior to implantation and were at 80% confluence on the day of the procedure. The cells were removed from the flask using standard cell culture technique. After counting, the cells were rinsed with PBS before being resuspended in serum-free, high-glucose DMEM at a cell density of 1 × 10^8^/ml. For liver inoculations, 25ul of this cell suspension is injected for a total number of cells of 2.5 × 10^6^. For subcutaneous inoculations, 100ul of this cell suspension is injected for a total number of cells of 1 × 10^7^. All procedures were conducted under anesthesia with isoflurane (1–4%) and oxygen (1–2 L) using a rodent anesthesia machine by tank induction followed by nose cone maintenance. Pedal withdrawal reflex was used to evaluate the depth of anesthesia for gas anesthesia before beginning any invasive procedure. Animals were placed on water circulating heating pads to maintain body temperature during surgeries, and the level anesthesia was continuously monitored throughout the surgeries. Both male and female mice were used, and male and female mice will by randomly allocated in a 1:1 ratio to experimental groups.

### MTPA and anti-PD-1 treatment

MTPA was performed as described previously^39–41^ and is predicated upon the strong affinity and retention of indocyanine green (ICG) in HCC. Briefly, animals were administered ICG 0.5 mg/kg via tail vein injection. Six hours following ICG injection, tumor specific hyperthermia was generated by a custom-designed system that was analogous to previously designed devices^39,41–43^. A 785 nm, 485 mW NIR laser (Edmund Optics) provided illumination and was coupled to a 2 mm collimating lens. Continuous thermometry was performed using an optical sensor (Fluoroptic, Lumasense, Santa Clara, CA) and an infrared camera (FLIR, Boston, MA). MTPA of orthotopic liver tumors was performed by direct visualization following mini-laparotomy and elaboration of the liver onto the skin surface. As the goal in these experiments was to stimulate tumor immunity rather than cause lethal hyperthermia for local tumor ablation, the target thermal dose was 42–45 °C for 5 min. Anti-PD1 antibody (clone RMP1-14, BioXcell, BE0146) was administered intraperitoneally at 250 μg per injection 24 h prior to MTPA (or sham surgery) followed by repeat administrations on post-operative days 3 and 7.

### Tumor growth measurements

For subcutaneous tumors, the tumor size was measured using a digital caliper and tumor volume was calculated using the formula V = 0.5*(height)*(width^2^).

### Tissue processing

Tissue processing was performed as previously described^14^. Briefly, the tumors were dissected and cut into small pieces with a scalpel. The tissue fragments were subject to enzymatic digestion in 3 volumes of PBS containing DNAse I 1 mg/mL (Roche cat# 11284932001), Collagenase D 1 mg/mL (Roche cat# 11088882001) and Dispase II 2.4 U/mL (Roche cat# 4942078001). After incubation at 37C for 30 min with gentle stirring, the lysate was washed with PBS+2% FBS and strained through a 40 µM nylon mesh. The red blood cells were then lysed with ammonium chloride buffer (Miltenyi cat #130-094-183). The cell suspension was washed and resuspended in PBS+2%FBS before determining the total number of cells obtained as well as their viability using Trypan blue. The leukocytes (i.e. CD45+ cells) were then purified using CD45 MicroBeads (Miltenyi cat#130-109-682) following the manufacturer’s indications. The purified cells were resuspended in PBS+2%FBS and counted as before.

### Flow cytometry

The cells were stained cells with the antibodies listed in Table 1. In brief, non-specific binding was first blocked by treating the samples with an anti-CD16/32 antibody (BioLegend, cat# 101302) at 1:200 at 4C for 5 min. The antibody dilutions were prepared in PBS + 2% FBS containing Live/Dead Fixable Aqua Dead cell stain (Invitrogen cat # L34966) at 1:1000. The cells were incubated with the baseline antibodies along with the specific surface markers for each panel during 30 min at 4C in the dark. For fixation and permeabilization prior to intracellular staining, the samples were treated with BD CytoFix/Cytoperm solution (BD cat # 554722) for 20 min at 4C. After 2 washes with BD Perm/Wash Buffer (BD cat # 554723), the intracellular stains were added to panels 1 through 3 as described in Table 1. After an incubation of 30 min at 4C in the dark, the cells were rinsed with the 1 × BD Perm/Wash Buffer, resuspended in PBS+2%FBS, and analyzed by flow cytometry with a Gallios 561 flow cytometer (Beckman Coulter). Please see the Supplemental Fig. 1 for a depiction of the gating strategy.

**Table 1:** Antibodies for leukocyte characterization via FACS.

Antibody	Clone	Fluorochrome	Catalog number	Dilution
**Baseline antibodies**				
CD45	30-F11	Alexa Fluor 700	BioLegend 103128	1:100
CD3	145-2C11	PerCP-Cy5.5	BioLegend 100328	1:12.5
**Panels 1 through 3**				
CD4	RM4-4	FITC	eBioscience/Invitrogen 11-0043-82	1:25
CD8a	53-6.7	APC-Cy7	BioLegend 100714	1:25
**Panel 1**				
Ki67*	SolA15	PE-Cy7	eBioscience/Invitrogen 25-5698-80	1:50
**Panel 2**				
Granzyme B*	GB11	Alexa Fluor 647	BD Pharmingen 560212	1:50
Interferon gamma*	XMG1.2	PE	BD Pharmingen 554412	1:50
**Panel 3**				
CD25	PC61.5	APC	eBioscience/Invitrogen 17-0251-81	1:50
FoxP3*	FJK-16 s	PE	eBioscience/Invitrogen 12-5773-80	1:50

*Intracellular stains.

### Immunohistochemistry

Flank tumors and tumor-bearing lungs were fixed in 10% formalin. Fixed tissues were processed, paraffin-embedded, sectioned, and stained by an institutional core service. The following antibodies were used for immunohistochemistry (IHC) staining: anti-CD3 (ab5690, Abcam, 1:100 dilution), anti-CD4 (ab183685, Abcam, 1:1000 dilution), anti-CD8 (ab209775, Abcam, 1:2000 dilution), anti-Ki67 (ab15580, Abcam), anti-FOXP3 (ab215206, Abcam, 1:250 dilution). Staining was performed according to the manufacturer’s instructions. Quantification of tumor staining was performed the stained sections using QuPath^44^ using previously published methods^45^.

### Genomic assays

All genomic analysis was performed within the R environment (R Foundation, version 4.0.2). Bulk RNA sequencing (RNAseq) was performed on liver and tumor tissue in the following manner. Tissue was harvested and was stored at − 80 °C until analysis was performed using the Illumina platform. Read alignment and transcript quantitation was performed by the kallisto package, and analysis was performed using the sleuth package^46^. Differentially expressed genes between the treatment groups was calculated using the DeSeq2 package. Gene set enrichment analysis of differentially expressed genes against the Hallmark gene set from MSigDB^47,48^ was performed on cirrhotic liver samples following cirrhosis induction compared to control liver samples using the fgsea package^49^. Likewise, the fgsea package was also used to determine the similarity between the HCA transcriptome and a previously published gene expression profile for HCC tumors classified by gain-of-function mutations of *CTNNB1* (β-catenin). To determine where on the spectrum of HCC aggressiveness the HCA-1 tumor model is located, we compared the HCA-1 transcriptome to publicly available human HCC genomic data in the following manner. Gene expression data from HCC tumors with known clinical Cancer of the Liver Italian Program (CLIP) scores were obtained from the Gene Expression Omnibus (GEO accession number GSE14520). The CLIP score is a validated clinical scoring system for human HCC with higher scores associated with worse clinical outcome^50^. The top 50 differentially expressed genes across these samples were identified. Unsupervised clustering of the human and mouse data was then performed after identifying the appropriate cross-species gene pairs to visualize similarities between the HCA-1 genome and human HCC. Given that the GEO data were acquired using the Affymetrix platform, while the mouse data were acquired using the Illumina platform, platform-specific bias and batch effects were accounted for using the ComBat-Seq package^51^.

For detailed characterization of the tumor immune microenvironment, single cell RNA sequencing (scRNAseq) was performed in the following manner. Orthotopic liver tumors were harvested following MTPA, anti-PD-1 therapy, combination MTPA+anti-PD-1 therapy, or sham surgery. CD45+ cells were isolated into single cell suspensions as previously described. The scRNAseq libraries were then prepared by the 10X Genomics Chromium single Cell Immune Profiling Solution based on the manufacturer’s recommendations using 10,000 purified leukocytes per sample. The cDNA of single cell transcriptomes was then sequenced using the Illumina platform (NextSeq 500). The data were analyzed using the Seurat package^52^. We first constructed an atlas of CD45+ cells that underwent scRNAseq. After identifying the major immune cell types, clusters were annotated based upon established transcriptional profiles^53^. Next, we subselected the clusters representing the T cell populations and characterized their phenotypic state using previously published gene sets^54^.

### Statistical considerations

All statistical analysis was performed within the R environment (R Foundation, version 4.0.2). Sample sizes and statistical methods were as described for the individual experiments. The non-parametric Mann–Whitney U-test was used to evaluate differences in the proportions across treatment groups. All reported P-values are two-sided, and the significance threshold was set at 5%.

## Supplementary Information

Supplementary Information.

## Data Availability

The authors declare that all data supporting the findings of this study are available within the paper and upon request. The datasets generated and/or analysed during the current study are available in the Gene Sequence Archive repository (accession #CRA009436).
