# Supplementary material for: Immune modulation by molecularly targeted photothermal ablation in a mouse model of advanced hepatocellular carcinoma and cirrhosis
Source: Sci Rep. 2022 Aug 24;12:14449. doi: 10.1038/s41598-022-15948-3 (PMC9402568; doi:10.1038/s41598-022-15948-3)

**Immune modulation by molecularly targeted photothermal ablation in a mouse model of advanced hepatocellular carcinoma and cirrhosis**

Nina M. Muñoz^1^, Crystal Dupuis^1^, Malea Williams^1^, Katherine Dixon^1^, Amanda McWatters^1^, Jie Zhang^2^, Swathi Pavuluri^1^, Arvind Rao^3^, Dan G. Duda^4^, Ahmed Kaseb^5^, Rahul A. Sheth^1^

^1^Department of Interventional Radiology, The University of Texas MD Anderson Cancer Center, Houston, TX, ^2^Department of Experimental Radiation Oncology, The University of Texas MD Anderson Cancer Center, Houston, TX, ^3^Department of Computational Medicine & Bioinformatics, University of Michigan, Ann Arbor, MI, ^4^Edwin. L. Steele Laboratories for Tumor Biology, Department of Radiation Oncology, Massachusetts General Hospital, Boston, MA, ^5^Department of Gastrointestinal Medical Oncology, The University of Texas MD Anderson Cancer Center, Houston, TX

**Supplemental Figures**

Supplementary Figure 1. FACS plots illustrating the expression of CD3, CD8, CD4, FoxP3, and Granzyme B on mouse HCC tumors. CD3 subsets were gated on CD45+ cells, and the CD4/CD8 subsets were gated on CD3+ cells.


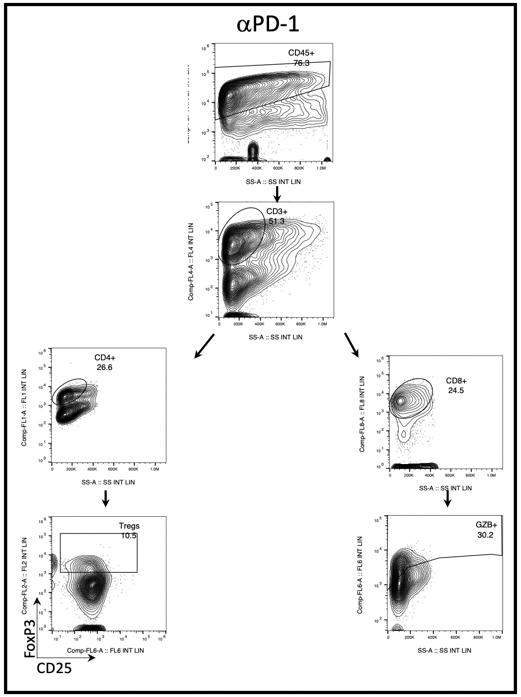

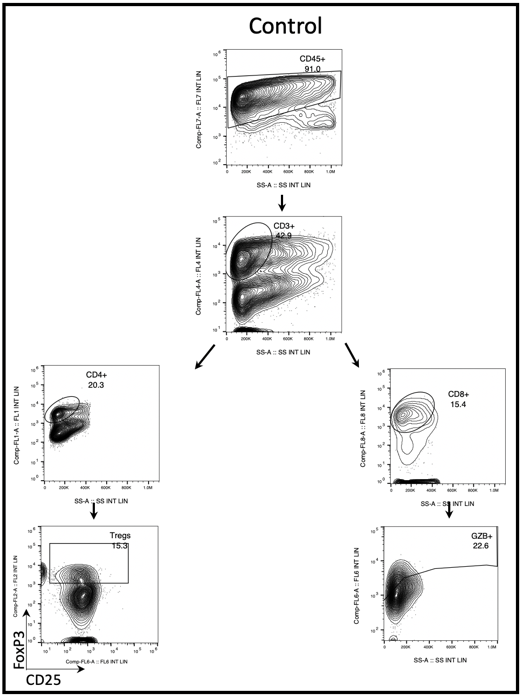

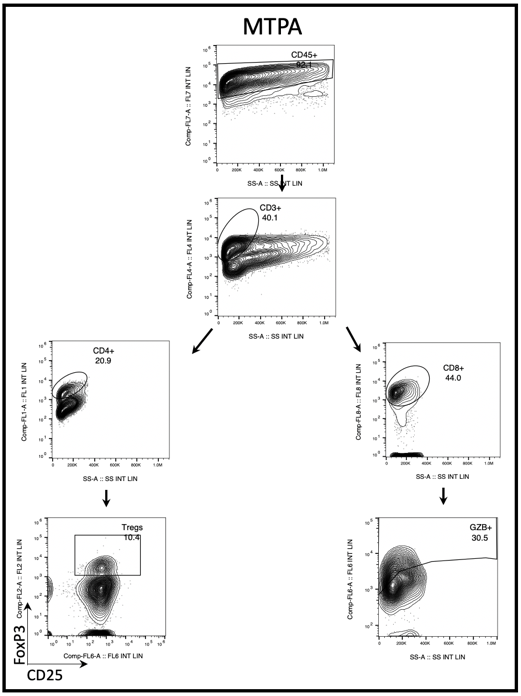


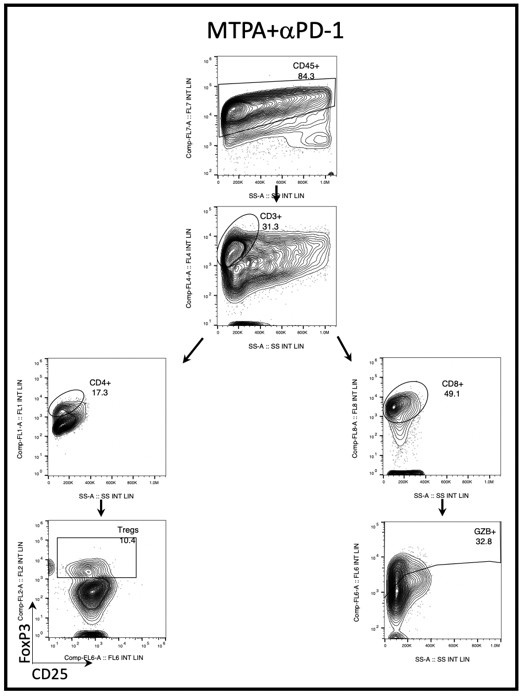

Supplement: Supplementary file 1 — Supplementary Information. [file 41598_2022_15948_MOESM1_ESM.docx]
